# Achieving Target Pressures with Combined Surgery: Primary Patchless Ahmed Valve Combined with Phacoemulsification *vs* Primary Phacotrabeculectomy

**DOI:** 10.5005/jp-journals-10008-1175

**Published:** 2015-01-15

**Authors:** Oscar Albis-Donado, Carmen C Sánchez-Noguera, Lorena Cárdenas-Gómez, Rafael Castañeda-Diez, Ravi Thomas, Félix Gil-Carrasco

**Affiliations:** Department of Glaucoma, Mexican Institute of Ophthalmology Queretaro, Mexico; Department of Glaucoma, Association for the Prevention of Blindness, Coyoacan, Mexico; Department of Glaucoma, Association for the Prevention of Blindness, Coyoacan, Mexico; Department of Glaucoma, Association for the Prevention of Blindness, Coyoacan, Mexico; Department of Glaucoma, Cataract, Clinical Epidemiology Queensland Eye Institute, Melbourne, Australia; Department of Glaucoma, Association for the Prevention of Blindness, Coyoacan, Mexico

**Keywords:** Phacoemulsification, Cataract, Glaucoma, Ahmed valve, Trabeculectomy, Combined surgery, Target pressure.

## Abstract

**Purpose:** To evaluate the ability of phacoemulsification combined with either primary trabeculectomy (PT) or primary Ahmed glaucoma valve implantation (PAVI) to achieve target intraocular pressures (TIOP) in adults with primary open angle glaucoma.

**Materials and methods:** Chart review of 214 adult patients operated between January 2002 and June 2008 with a minimum follow-up of 6 months. Group 1 comprised 181 eyes of 166 patients undergoing PT while group 2 included 50 eyes of 49 patients in combination with primary AVI. Target lOPs were pre-determined for each patient and success was defined as an IOP at or lower than target with or without medications. An IOP above target, loss of light perception or need for additional procedures to lower IOP were considered a failure.

**Results:** Mean preoperative IOP was 17.2 mm Hg in group 1 and 17.3 in group 2. Mean postoperative IOPs were 10.2 and 9.2 on day 1, 12.2 and 11.6 at year 1, and 10.7 in both groups at year 5. Survival rates in groups 1 and 2 were 96.7 *vs* 96% at 6 months, 89 *vs* 96% at 12 months, 83.5 *vs* 96% at 24 months and 79.4 *vs* 89.1% at 36, 48 and 72 months. Transient bleb leaks were more frequent in group 1 (26 eyes, 14.4 *vs* 0%, p = 0.001) and transient choroidal detachments were more frequent in group 2 (7 eyes, 3.9 *vs* 6 eyes, 12%, p = 0.038).

**Conclusion:** Midterm results for achieving target pressures using combined phacoemulsification with either PT or PAVI are comparable. The profile of complications is different for the two procedures.

**How to cite this article:** Albis-Donado O, Sánchez-Noguera CC, Cárdenas-Gómez L, Castañeda-Diez R, Thomas R, Gil-Carrasco F. Achieving Target Pressures with Combined Surgery: Primary Patchless Ahmed Valve Combined with Phacoemulsification *vs* Primary Phacotrabeculectomy. J Curr Glaucoma Pract 2015;9(1):6-11.

## INTRODUCTION

Combining glaucoma and cataract surgery for coexistent open angle glaucoma (OAG) and visually significant cataract provides an opportunity for timely visual rehabilitation as well as control of intraocular pressure (IOP).^1-3^

Noben et al and others^4,5^ compared combined phacoemulsification and trabeculectomy (PT) *vs* trabeculectomy alone as a primary procedure, and reported a better 1-year IOP reduction with trabeculectomy alone. Two systematic reviews by Jampel and Friedman et al found that combined surgeries were better than cataract surgery alone for IOP control and that using phacoemulsification rather than nuclear expression resulted in better IOP control.^6,7^

Implantation of glaucoma drainage devices (GDDs), usually reserved for cases with failed conventional procedures or conditions like neovascular glaucoma and uvei-tis that are predisposed to failure with standard filtering surgery, are increasingly being considered earlier in the course of the disease.^8-11^ The few published studies on combined cataract and GDDs report encouraging results despite inclusion of refractory glaucomas in the series.^12,13^

Target IOPs are rarely reported as an endpoint for surgical glaucoma trials,^14^ but since they are defined as the IOP level or range at which no further glaucoma damage occurs,^15^ it makes sense to try to report them as a goal for glaucoma surgeries.

Motivated by encouraging results with implants in pseudophakic and aphakic eyes, surgeons at the Aso-ciacion Para Evitar la Ceguera en Mexico (Association for the Prevention of Blindness in Mexico) have combined primary Ahmed glaucoma valves with cataract surgery since 2000.^16^ We also perform primary phacotrabeculec-tomy for such cases and both procedures are taught to residents. This provided an opportunity to compare achievement of target IOP control in cases undergoing PT or PAVI as a primary procedure in a routine situation.

## MATERIALS AND METHODS

Charts of the 214 patients over 18 years of age operated at the ‘Asociacion Para Evitar la Ceguera en Mexico’ with either phacotrabeculectomy (group 1) or Phaco and Ahmed glaucoma valve (group 2) between January 2002 and June 2008 were reviewed. Each patient had signed a standard consent form and the study was approved by our institution’s Ethics Committee for retrospective analysis.

Adult, Mexican Mestizos with primary, pseudoexfo-liation or pigmentary open-angle glaucomas not reaching target IOP and in need of cataract surgery were included. Inclusion required a minimum of 6 months post-surgical follow-up. Patients with other types of glaucoma, retinal disease, nystagmus, previous conjunctival, retinal, ref rac-tive or other corneal surgeries were excluded.

Surgery was performed by both faculty and fellows. There were no specific instructions or criteria to decide between the two procedures; the choice was left to the consultant surgeon. While the type of procedure used was decided by the surgeon, in our department valves were generally preferred in moderate and severe cases, and/or when it was felt a trabeculectomy had a higher risk of failure, higher chance of infection for any reason (e.g. manual labor in the fields) and in patients judged unlikely to comply with follow-up visits.

Phacotrabeculectomy was initiated with the initial steps of trabeculectomy. The choice of limbus *vs* fornix-based conjunctival flap and single *vs* twin site was made by the surgeon. For twin site surgery, following construction of the scleral flap, a separate corneal incision was made for the phacoemulsification. Once the IOL was implanted, viscoelastic was left in the anterior chamber, the trabeculectomy was completed and a peripheral iri-dectomy was performed. Viscoelastic was removed and closure of the scleral flap was titrated to allow ooze of aqueous either spontaneously or on gentle pressure. Conjunctiva was closed using 10-0 nylon, a continuous running suture was used for limbus-based trabeculectomies and episcleral mattress plus running sutures as needed were used for fornix-based trabeculectomies.

The use of mitomycin for phaco trab is not routine in the department and all such operations were done without mitomycin. The decision and timing of suture lysis was made by the surgeon.

The AGV S2 model (New World Medical, California, US) was used for all PAVI procedures. The surgical technique used in these cases has been described in detail elsewhere and does not involve use of a scleral patch graft.^17-19^ In brief, a fornix-based conjunctival flap was created in the superotemporal quadrant and the Ahmed plate was sutured to the scleral bed 8 to 10 mm behind the limbus with 7-0 silk. A scleral tunnel initiated 3 to 4 mm from the limbus was constructed using a 23 G needle, bent in a ‘Z’ formation to avoid interference from the eyelids, brow or lid speculum.^20^ The direction of the needle was abruptly changed at the limbus to make the anterior chamber entry parallel to the iris. The needle was mounted on a viscoelastic syringe, so the anterior chamber could be reformed after needle entry into the anterior chamber. The AGV silicone tube was then trimmed to create a 30 to 45° bevel and inserted through the tunnel into the anterior chamber. Phacoemulsification was then performed, according to surgeon’s preference. The conjunctiva was closed using the same 7-0 silk. The postoperative regimen in both the groups included prednisolone acetate 1% every 2 hours during the daytime in the first month and tapered slowly over 3 months. Antibiotic drops (ciprofloxacine, moxifloxacine or gatifloxacine) four times a day were used for 2 weeks, along with a cycloplegic agent for the first month in all patients.

Postoperatively, patients were examined at days 1, 3, 7, 14, 30 and months 2, 3, 6, 12, and at least once a year thereafter.

Target IOPs were determined for each patient depending on the mean deviation of the last visual fields before surgery. Based on the mean deviation, the eyes were arbitrarily divided into three groups: -5.99 dB or better (mild glaucomas), between -6 and -11.99 dB (moderate glaucomas) and those with MD worse than -12dB (severe glaucomas) and had their target IOPs set at 16, 14 and 12 mm Hg respectively.

Success was defined as an IOP at or lower than target with or without medications. Inability to achieve target IOP with medications, loss of light perception and/or need for additional procedures to lower IOP was considered a failure.

Visual acuity was measured by a technician using Snellen charts, and best-corrected visual acuity was recorded. Slit-lamp examination was performed and IOPs measured using Goldmann applanation tonometry between 8 am and 1 pm by the surgeon, a fellow or a resident. Fields were performed as per our clinical routine. Each active ingredient of single or combined drops or oral formulations was counted as a separate medication.

Statistical analysis was performed using SPSS version 19. Means for continuous variables were compared among the different groups using analysis of variance (ANOVA). Associations between categorical variables were studied with Chi-square test; Fisher’s exact test was used when fewer than 5 cases were present at a given category. Relative risk and confidence intervals were calculated for these data. Multivariate models were constructed to determine the effect of risk factors on the final survival rate. As both the eyes of 16 patients were included in the analysis, generalized estimating equations were used for multivariate analysis of risk factors for failure, adjusting for dependencies when both eyes of the same patient had been operated, repeated measures and missing data. Forward stepwise selection of covariates and factors significant in the simple analysis was used for the final multivariate model. Survival analysis was performed using the Kaplan-Meier life-Table method, using the log-rank test for comparing survival times between groups.

## RESULTS

Group 1 comprised 181 eyes of 166 patients while group 2 consisted of 50 eyes of 49 patients; 68% were female. Mean age was 69.1 years for group 1 and 72.8 for group 2; this was statistically different but did not affect results in either uni or multivariable analysis (Table 1).

**Graph 1:**
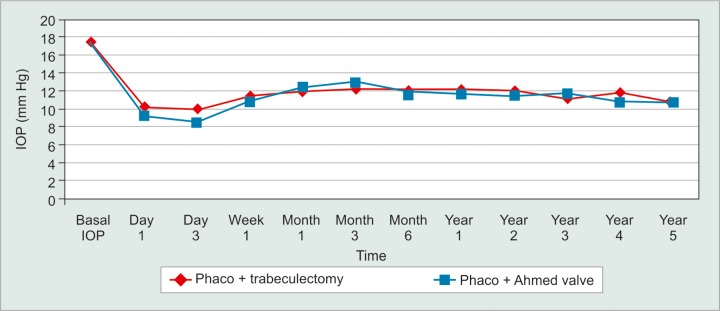
Mean IOP according to technique during follow-up. IOP was similar at all time-points

One hundred and forty two eyes had severe glaucoma (61.7%), 55 had moderate glaucoma (23.9%) and 33 had mild glaucoma (14.3%). Mean preoperative IOP was 17.2 mm Hg in group 1 and 17.3 in group 2. Mean target IOP for all eyes was 13.05 mm Hg. The IOP was lowered to 10.2 and 9.2 by day 1, 12.2 and 11.6 by year 1, and 10.7 and 10.7 by year 5. Differences were not statistically significant at any time-point (Graph 1). Mean last recorded IOP was statistically lower for group 2 (12.2 *vs* 11.1, p = 0.025, 95% Ci 0.14 to 2.13).

Survival rates for maintaining target IOP in groups 1 *vs* 2 eyes were 96.7 *vs* 96% at 6 months, 89 *vs* 96% at 12 months, 83.5 *vs* 96% at 24 months and 79.4 *vs* 89.1% at 36, 48 and 72 months, the differences were not significant and confidence intervals for the difference ranged from -0.35 to 0.51% at each time-point. After 36 months of follow-up, the number of eyes remaining was less than half of the original cohort, so most of our comments will be limited to within that time period.

**Graph 2:**
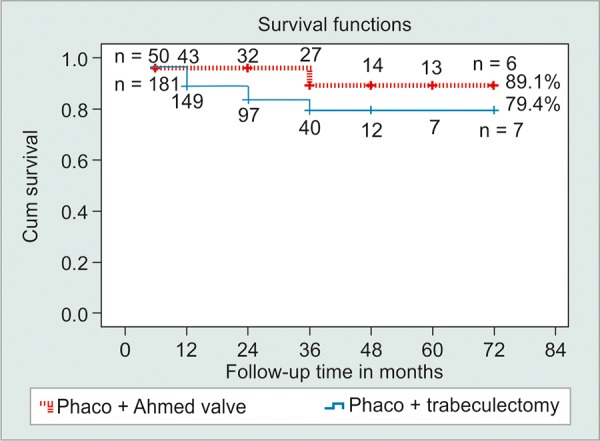
Kaplan-Meier survival curve for phaco-Ahmed *vs* phacotrabeculectomy, the difference between the 2 groups is not statistically significant. Numbers in boxes represent the number of eyes still under observation at the corresponding follow-up time

**Table 1:** **Table 1:** Demographic data by group

*Characteristic*		*Phaco-trab* * (n = 181)*		*Phaco-AVI* *(n = 50)*		*p*	
Age, year (mean ± SD)		69.2 ± 8.8		72.8 ± 8.3		0.010	
Gender, female, N (%)		122 (67.4%)		35 (70%)		0.434	
Eye OD, N (%)		97 (53.6%)		20 (40%)		0.061	
Follow-up months (mean ± SD)		22.1 ± 15.3		34.4 ± 23		< 0.001	
Preoperative IOP mm Hg (mean ± SD)		17.3 ± 5.5		17.3 ± 4.5		0.943	
Target IOP mm Hg (mean ± SD)		13.1 ± 1.5		12.8 ± 1.3		0.249	
Preoperative visual field damage severity (N, %)						0.417	
• Mild		30 (16.6%)		4 (8%)			
• Moderate		42 (23.2%)		13 (26%)			
• Severe		109 (60.2%)		33 (66%)			

Mean survival time (as determined by target IOP) for PT was 61.1 months (95% CI 57.17-65.01); for PAVI this was 66.9 months (95% CI 62.12-71.66); the difference was not statistically significant (Graph 2).

**Table 2:** **Table 2:** Number of eyes reaching their corresponding target IOP without medications in each group, and mean number of medications needed in each group at last follow-up. None of the differences were statistically significant

		*Target IOP with no medications*						*Total eyes reaching target with no medications*			
		*12*		*14*		*16*				*Mean number of medications*	
Phaco-trab		27 (24.8% of 109 eyes)		11 (26.2% of 42 eyes)		5 (17.2% of 29 eyes)		43 (23.9% of 180 eyes)		1.60	
Phaco AVI		8 (24.2% of 33 eyes)		3 (23.1% of 13 eyes)		1 (25% of 4 eyes)		12 (24% of 50 eyes)		1.58	
Mean number of medications		1.69		1.45		1.45					

**Table 3:** **Table 3:** Summary of postoperative complications by surgical technique (CRVO: central retinal vein occlusion)

*Complications*		*Phaco-trab* *(n = 181)*		*Phaco-AVI* *(n = 50)*		*p*	
Transient choroidal detachment (N, %)		7 (3.9%)		6 (12%)		0.038	
Bleb leak (N, %)		26 (14.4%)		0		<0.001	
Transient corneal edema (N, %)		22 (12.2%)		2 (4%)		0.071	
Transient flat anterior chamber (N, %)		1 (0.6%)		0		0.784	
CRVO (N, %)		1 (0.6%)		0		0.784	
		0		0			
		0		0			
		0		0			
Tube extrusion/exposure (N,%)		NA		0			
Diplopia (N, %)		0		0			

NA: Not available

The number of medications needed to control IOP during the full follow-up period were similar at all time-points except month 6 (0.92 in group 1 *vs* 0.58 in group 2 (p = 0.023), and month 12 (1.41 in group 1 *vs* 0.83 in group 2 (p = 0.002). The number of eyes reaching target without medications and the mean number of medications used in each target group are shown in Table 2.

Thirty-three of 181 eyes (18.23%) in group 1 underwent suturelysis and 44 (24.31%) required needling with 5-fluorouracil as decided by the surgeon. These two variables had no effect on the final success status or the number of bleb leaks. Eyes in group 2 did not receive similar interventions.

As shown in Table 3, transient bleb leaks were more frequent in group 1 (26 eyes, 14.4% *vs* 0%) and transient choroidal detachments were more frequent in group 2 (7 eyes, 3.9% in group 1 *vs* 6 eyes, 12% in group 2, p = 0.038)

## DISCUSSION

The generally accepted initial approach for OAG patients in need of filtering surgery is trabeculectomy. Different techniques of combined surgeries have been described to control IOP and deal with any clinically significant cataract that may be present.^21^ Surgical times and complications vary widely depending on surgeon experience, technology available and precautions taken during the procedure.

Using target IOP as an endpoint should have the potential to better predict if any kind of surgery can better achieve our ultimate goal, to prevent further glaucoma damage.^15^ A swift comparison of visual field progression of the CIGTS patients, which used target IOP as the therapeutic target (37% mean IOP reduction),^22^ and the EMGT patients (25% mean IOP reduction)^23^ shows a large difference in progression (21.3% surgery and 25.5% medicine over 8 years *vs* 59% over 8 years). Target IOPs are also a much stricter outcome than the usual 21 mm Hg cut off or even the more recently advocated thresholds of 18 or 15 mm Hg.^24-28^

The IOP reduction achieved using phacoemulsification combined with Ahmed valve glaucoma implants was comparable to that obtained using phacoemulsification with trabeculectomy. As far as IOP lowering medications are concerned the results tend to favor the AGV option but are unlikely to be clinically significant. While the numbers are small (and statistically not significant) the Kaplan-Meier curves suggest AGV has potentially longer survival. The number needed to treat (NNT) is 10 (CI 95% -27 to 5, equivalent to an absolute risk reduction (ARR) ranging from -0.036 to 0.185). If we consider the absolute success and failure rates, without taking time into account, the NNT is 16 (CI 95% -19 to 7, equivalent to ARR -0.053 to 0.140). And while we did find a significant difference in last recorded IOP between the groups, the magnitude of this divergence is small and does not take into account the differential follow-up times.

Phacotrabs are generally performed using MMC modulation. REF, However, we did not use MMC and it is possible that such supplementation of phacotrabe-culectomy and/or the use of post operative 5 fluorouracil in all eyes might have resulted in better IOP control in this group. The IOP levels and number of early bleb-leaks in this group are comparable to previous reports.^29-31^ On the other hand late onset bleb-leaks, more commonly seen with the use of antifibrotic agents and a known risk factor for bleb-related infections,^32^ were not found in our series.^33^

Since the risk of early bleb leaks is higher with trabeculectomies^29^ and in our series with phacotrabeculectomies, the need for closer monitoring may justify a tube when the patient lives far or will have poor compliance with follow-up visits.^34^

Other complications seem to have a lower incidence than what is published; two deserve special attention. The lack of cases with hyphema is probably related to the retrospective nature of the present study obtaining data via a chart review. Small hyphemas were probably not mentioned in the chart. On the other hand, the lack of tube extrusions and exposures is actually closer to truth, since any such complication would need a surgical procedure that would necessarily be recorded at the chart; our patchless technique has been reported to have a low rate of tube extrusions/exposure.^19,35^

Since this was a retrospective study, it was not possible to objectively monitor the precise time each procedure took. In our hands Ahmed valves using the patchless technique can be implanted in 30 to 40 minutes for residents in their first surgeries to 10 to 15 minutes for attending surgeons. Once implanted, a postoperative IOP course is reasonably predictable and permits the patient to return to their normal activities earlier than a trabeculectomy. Also an eye with an Ahmed valve can more easily resist trauma, is less prone to infections, has less issues with swimming or contact lenses, and has similar induction of astigmatism.^36^

This report suffers from the limitations of a chart review and the loss to follow-up. It also reflects practice in a teaching hospital with both consultants and fellows with differing skill levels performing surgery.

To replace a standard technique, the new procedure should have better results, fewer complications or should be cheaper. Our uncontrolled preliminary results with an NNT of 10 suggest that combined Ahmed valve and phacoemulsification can be a potential alternative for eyes with OAG needing primary filtering surgery plus cataract removal. The AGV does add to the price of surgery and it remains to be seen if long-term results will support the potential advantages of shorter time, less postoperative monitoring and the cost benefit ratio.
